# Investigation and Analysis of Eye Discomfort Caused by Video Display Terminal Use Among Medical Students Studying at High-Altitude Regions

**DOI:** 10.3389/fpubh.2022.900539

**Published:** 2022-05-12

**Authors:** Bingjie Liu, Shanshan Jiang, Zuyou Li, Yao Wang, Daijiao Zhou, Zhen Chen

**Affiliations:** ^1^The Affiliated Hospital of Kunming University of Science and Technology, Kunming, China; ^2^Department of Ophthalmology, The First People's Hospital of Yunnan Province, Kunming, China; ^3^Dali University, Dali, China

**Keywords:** video display terminal (VDT), medical students, high-altitude regions, dry eye, tear film distribution

## Abstract

**Objective:**

To investigate the use of video display terminal (VDT) in high-altitude regions by medical students and the resulted risk factors of eye discomfort.

**Methods:**

A cross-sectional study was conducted in 686 medical students randomly selected from high-altitude regions, Kunming, Yunnan Province. The prevalence of video display terminal syndrome (VDTs) and related risk factors in medical students were analyzed by instructing students to fill in the eye discomfort symptom questionnaire [Ocular Surface Disease Index (OSDI) scale].

**Results:**

There were 686 participants in this study, including 213 males (31.0%) and 473 females (69.0%). The results of questionnaire survey showed that 184 patients (26.8%) didn't have eye discomfort symptoms and 502 patients (73.2%) had eye discomfort symptoms. The comparison of demographic and ocular characteristics of eye discomfort symptoms group and no eye discomfort symptoms group showed that medical students in the group with ocular discomfort symptoms had longer total duration of video terminal use, longer duration of each VDT use, higher total scores of ocular discomfort symptom scores, and a higher percentage of the ocular discomfort severity group, all with statistically significant differences. There was no significant difference in the remaining parameters between the two groups of participants. The results of multivariate logistic regression model analysis showed that the relationship between total sleep time, total duration of VDT use and eye discomfort symptom score was statistically significant (*P* < 0.05). Besides, ordinal logistic regression was applied further to investigate related factors of the severity of eye discomfort. The model demonstrated that total sleep duration and total VDT using duration were significantly associated with the severity of eye discomfort symptom (*P* < 0.05).The severity of eye discomfort symptom was significantly negatively related to the increased total sleep duration and it was significantly positively related to the increased VDT use duration.

**Conclusion:**

The prevalence of VDTs is high in medical students at high-altitude regions. The longer total duration of daily use of VDT and shorter sleep duration are risk factors associated with VDTs. The severity of eye discomfort symptom was significantly negatively related to the increased total sleep duration and it was significantly positively related to the increased total VDT using duration.

## Introduction

With the gradual advancement of social informatization and the widespread popularization of electronic products such as mobile phones, tablets, and computers, video display terminal (VDT) has infiltrated into every part of our life, study, and work, bringing various conveniences to our lives and improving our work efficiency, while video display terminal syndromes (VDTs) has become more and more common (1). These related eye diseases caused by frequent use of VDT are collectively referred to as VDTs, a clinical syndrome, which is mainly manifested as dry eyes, eye astringent, eye redness, eye pain, asthenopia, dizziness, blurred vision, diplopia, and other symptoms in some users after operating the computer or staring screen for a long time and at close distance, while some users are accompanied by different degrees of systemic symptoms such as shoulder and neck pain (2, 3).

The International Dry Eye Working Group in 2007 defined dry eye as a multifactorial ocular surface disease characterized by loss of intraocular homeostasis with tear film instability and hyperosmolarity, ocular surface inflammation and injury, and neurosensory abnormalities as ocular symptoms (4). Epidemiological research studies have shown that common risk factors for dry eye include female, age-related, prolonged contact-lens wearing, long-term use of hormones, irrational use of eye drops, vitamin A deficiency, prolonged use of VDT, excessive eye use, and so on (5). Currently, there is no standardized criteria for the diagnosis of VDTs as an important component of dry eye, but the presence of subjective symptoms of ocular discomfort is one of the necessary conditions for diagnosis (6, 7). The OSDI scale is the current common scale used internationally to assess the subjective symptoms of ocular discomfort symptoms (8). The scale consists of 12 questions, including the assessment of ocular symptoms, visual function and environmental factors and the severity of eye discomfort symptoms is classified and grouped according to different total scores: <6 as no eye discomfort symptoms, 6–20 as mild eye discomfort symptoms, 20–45 as moderate eye discomfort symptoms, and >45 as severe eye discomfort symptoms (9).

With the popularity of electronic medical records, the frequency of exposure to VDT among medical students enrolled in colleges and universities has also increased tremendously in proportion. It has been observed during internship process and follow-up clinic visits that many medical undergraduates have eye complaints, and there are relevant literature reports that symptomatic dry eye may be prevalent among medical students (10). Therefore, medical students in school were selected as the subjects of this study.

At the same time, high-altitude regions also affect the appearance of eye discomfort symptoms to varying degrees due to their very unique characteristics of natural environment and geographical conditions (11). Kunming, Yunnan Province in China, as one of the high-altitude areas, has an average altitude of about 1,890 m, an average annual temperature of about 12–22°C, an average annual relative humidity of about 62%, an annual total cloudiness of about 50%, an annual precipitation of about 900 mm, an annual average wind of 4 m/s, and an annual sunshine duration of about 2,500 h. In contrast, Chinese coastal cities such as Nanjing has an average altitude of 20–30 m, an average annual temperature of 13–22°C, an average annual relative humidity of about 72%, an annual total cloudiness of about 70%, an annual precipitation of about 1,200 mm, an annual average wind of up to 3 m/s, and an annual sunshine duration of about 1,500 h. Compared with coastal cities, the unique natural environment of high altitude areas, including long sunshine hours, low precipitation, strong wind and dryness, may have adverse effects on the human body, especially the eyes. In particular, the increase in altitude and the consequent increase in UV intensity is an important factor in aggravating eye discomfort (12, 13).

In view of the above reasons, 686 medical students majoring in medicine at Kunming University of Science and Technology and Kunming Medical University in Yunnan Province were selected as the survey subjects in this study and the ocular discomfort symptom questionnaire was filled out so as to analyze the prevalence and related risk factors of VDTs after using VDT in medical students at high-altitude regions. The survey results are reported as follows.

## Informations and Methods

### Objects

A total of 686 medical students were randomly selected from colleges and universities in Kunming, Yunnan Province, including a total of 213 males and 473 females. The average age was (20.86 ± 4.33) years.

### Inspection Method

Students were instructed to complete the eye discomfort symptom questionnaire under a uniform measurement standard. The questionnaire results were divided into 4 groups according to the total score: no eye discomfort symptoms: 0–5 points; mild eye discomfort symptoms: 6–20 points; moderate eye discomfort symptoms: 21–45 points; and severe eye discomfort symptoms: >45 points. In this study, the risk factors of VDTs in medical students in colleges and universities were further studied by adding the four questions of whether refractive surgery or other eye surgery was performed, whether contact lenses were worn for more than 2 h per day, the length of daily sleep, and the total duration of daily VDT use and the total duration of VDT use on the basis of the OSDI scale.

### Statistical Methods

The statistical software R (Version 4.1.1; R Core Team, 2021) was used for statistical description and inference. Quantitative data were expressed as mean and standard deviation; qualitative data were expressed as frequency and percentage. Comparison among groups of quantitative data were conducted using independent sample *t*-test and test inspection for qualitative data. In addition, multivariate logistic regression models were used to further explore the influencing factors of the eye discomfort symptom score. Ordinal logistic regression was applied further to investigate related factors of the severity of eye discomfort. The test level was α = 0.05, and the difference was considered statistically significant when *P* < 0.05.

## Results

### General Clinical Characterization

A total of 686 subjects meeting the criteria were included in this study. The average age was 20.86 ± 4.33 years, including 213 (31.0%) males and 473 (69.0%) females. There were 184 patients (26.8%) in the group without eye discomfort symptoms, while 502 patients (73.2%) showed eye discomfort symptoms, as shown in Table 1.

**Table 1:** Description of the general clinical characteristics of the study subjects.

**Variable**	***N*(%)$\sqrt{x\pm s}$**
**Gender**
Male	213 (31.0)
Female	473 (69.0)
Age	20.86 ± 4.33
**Perform refractive surgery or other eye surgery**
Yes	29 (4.2)
No	657 (95.8)
**Wearing contact lenses for more than 2 h per day**
Yes	67 (9.8)
No	619 (90.2)
**Total sleep time (h)**
5	17 (2.5)
6	136 (19.8)
7	320 (46.6)
8	213 (31.0)
Total duration of daily VDT use (h)	3.77 ± 2.43
Duration of each VDT use (h)	2.60 ± 1.34
Total score	10.00 (4.00, 22.00)
**Degree of eye discomfort**
Mild eye discomfort	311 (45.3)
No eye discomfort	184 (26.8)
Moderate eye discomfort	170 (24.8)
Severe eye discomfort	21 (3.1)
**Eye discomfort**
Yes	502 (73.2)
No	184 (26.8)

### Demographic and Ocular Characteristics, Eye Discomfort Symptoms Group vs. No Eye Discomfort Symptoms Group

The comparison of demographic and ocular characteristics between the group with ocular discomfort symptoms and the group without ocular discomfort symptoms showed statistically significant differences in the two groups of indicators: total sleep time, and total duration of daily VDT use. Shorter daily sleep duration and longer total duration of daily VDT use may have contributed to the presence of VDTs. The differences in the remaining indicators including gender, age, whether refractive surgery or other eye surgery was performed, and whether corneal contact lenses were worn for more than 2 h per day were not statistically significant and may not affect the occurrence of VDTs.As shown in Table 2.

**Table 2:** Single factor comparison eye discomfort symptoms group vs. no eye discomfort symptoms group.

**Variables**	**Eye discomfort symptoms group** **(*N* = 502)**	**No Eye discomfort symptoms group** **(*N* = 184)**	***t/Z*/x^2^**	***P***
**Gender**			3.379	0.066
Male	146 (29.1)	67 (36.4)		
Female	356 (70.9)	117 (63.6)		
**Age**	20.82 ± 4.30	20.97 ± 4.43	−0.402	0.688
**Perform refractive surgery or other eye surgery**			0.273	0.601
Yes	20 (4.0)	9 (4.9)		
No	482 (96.0)	175 (95.1)		
**Wearing contact lenses for more than 2 h per day**			0.327	0.567
Yes	51 (10.2)	16 (8.7)		
No	451 (89.8)	168 (91.3)		
**Total sleep time (h)**			8.028	**0.045**
5	12 (2.4)	5 (2.7)		
6	103 (20.5)	33 (17.9)		
7	246 (49.0)	74 (40.2)		
8	141 (28.1)	72 (39.1)		
**Total duration of daily VDT use (h)**	4.01 ± 2.45	3.12 ± 2.26	4.446	**<0.001**
**Duration of each VDT use (h)**	2.70 ± 1.36	2.35 ± 1.25	3.164	**0.002**
**Total score**	16.00 (10.00, 24.00)	2.00 (0.00, 4.00)	−20.130	**<0.001**
**Degree of eye discomfort**			686.000	**<0.001**
Mild eye discomfort	311 (62.0)	0 (0.0)		
No eye discomfort	0 (0.0)	184 (100.0)		
Moderate eye discomfort	170 (33.9)	0 (0.0)		
Severe eye discomfort	21 (4.2)	0 (0.0)		

*The bold values indicate statistically significant values of p < 0.05*.

### Multivariate Logistic Regression Model of Factors Associated With Eye Discomfort Symptom Score

**Figure 1:**
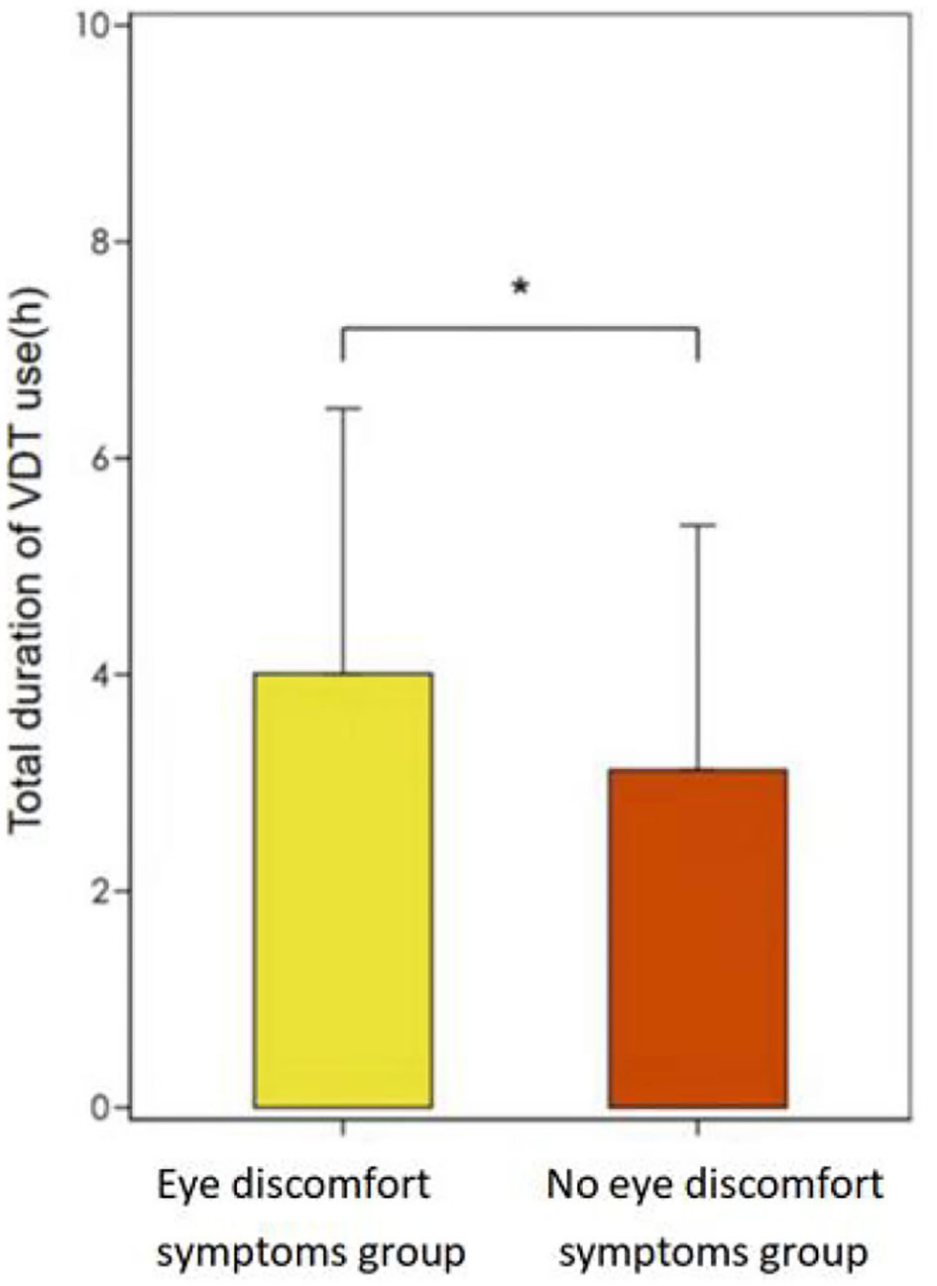
Total duration of VDT use, eye discomfort symptoms group vs. no eye discomfort symptoms group. *means the difference between the two groups has statistically significant.

**Figure 2:**
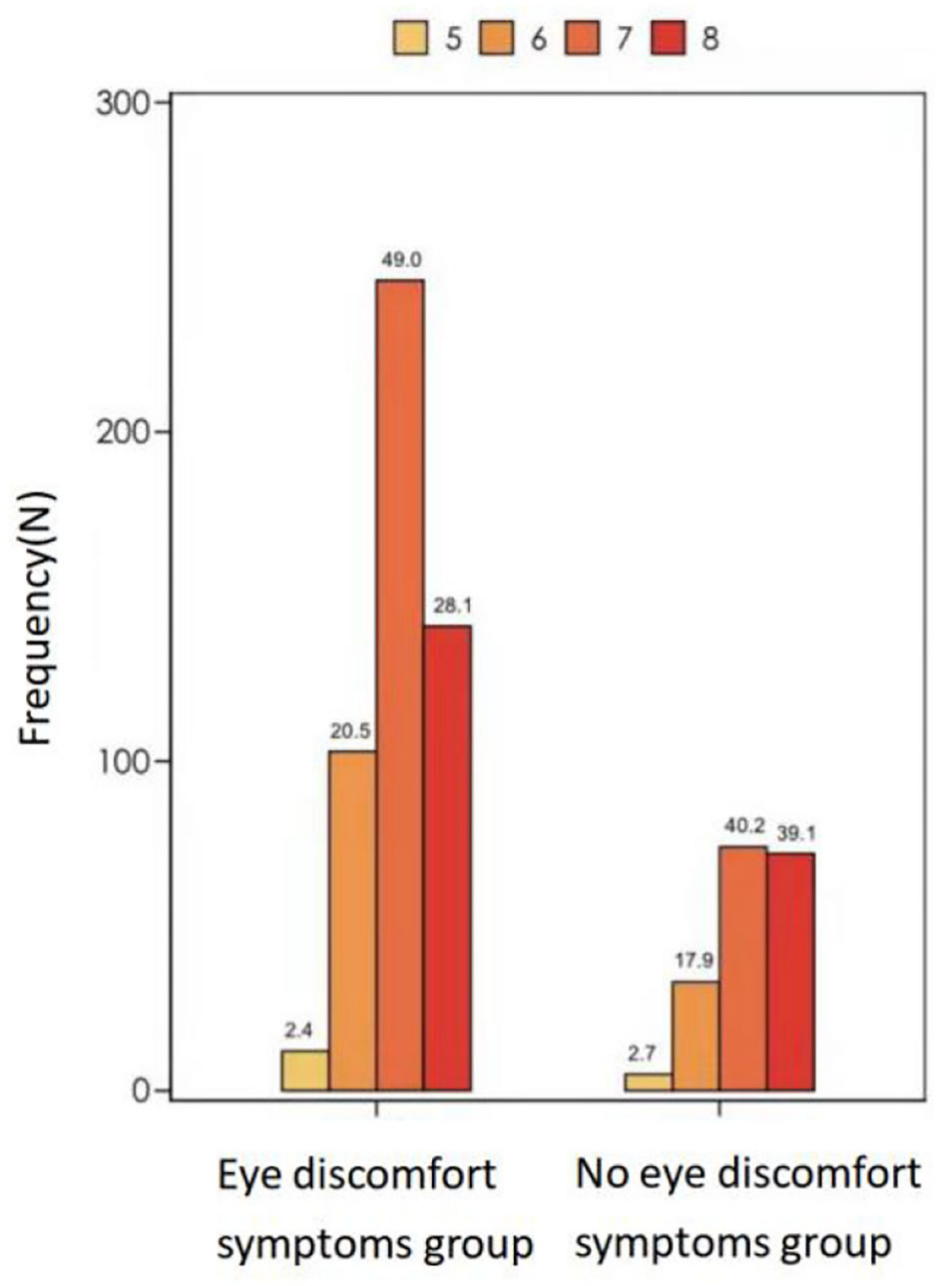
Total sleep time, eye discomfort symptoms group vs. no eye discomfort symptoms group.

The above variables were further included in a multifactorial logistic regression model to create a multifactorial regression model of patients' eye discomfort symptom scores. The analysis showed that (1) Total daily sleep time: OR_totalsleeptime_ = 0.768, *P* = 0.042, indicating that for every 1 h increase in the patients' total daily sleep time, their likelihood of developing eye discomfort symptoms decreased by 23%; (2) Total duration of daily VDT use: OR_TotaldurationofdailyVDTuse_ = 1.162, *P* = 0.001, indicating that for every 1 h decrease in the patients' daily reduction in total duration of VDT, their likelihood of developing eye discomfort symptoms decreased by 16%. Total daily sleep time and total duration of daily VDT use were risk factors associated with dry eye symptom scores, and the effects of the remaining factors were not statistically significant. We can see the Table 3, Figures 1, 2 for the details.

**Table 3:** Logistic regression model of factors associated with eye discomfort symptom score.

**Variables**	**B**	**SE**	**Z**	***P***	**OR**	**LCL**	**HCL**
Intercept	0.283	1.137	0.249	0.803	1.328	0.143	12.333
Gender	0.291	0.189	1.539	0.124	1.338	0.923	1.939
Age	−0.005	0.02	−0.234	0.815	0.995	0.958	1.034
Perform refractive surgery or other eye surgery	0.296	0.434	0.682	0.495	1.345	0.574	3.152
Wearing contact lenses more than 2 h a day	−0.143	0.314	−0.455	0.649	0.867	0.468	1.604
**Total sleep time**	−0.241	0.118	−2.036	**0.042**	0.786	0.623	0.991
**Total duration of VDT use**	0.150	0.045	3.304	**0.001**	1.162	1.063	1.270
Duration of each VDT use	0.095	0.08	1.187	0.235	1.099	0.940	1.285

*The bold values indicate statistically significant values of p < 0.05*.

## Ordinal Logistic Regression for Related Factors for the Severity of Eye Discomfort Symptoms

Ordinal logistic regression was applied further to investigate related factors of the severity of eye discomforts. The model demonstrated that total sleep duration and total duration of daily VDT use were significantly associated with the severity of eye discomfort symptoms. OR_Totalsleepduration_ = 0.776, *P* < 0.05, demonstrating that the severity of eye discomfort symptoms was significantly negatively related to the increased total sleep duration. OR_TotaldurationofdailyVDTuse_ = 1.205, *P* < 0.001, indicating that the severity of eye discomfort symptoms was significantly positively related to the increased total duration of daily VDT use, Table 4.

**Table 4:** Ordinal logistic regression for related factors for the severity of eye discomfort symptoms.

**Variables**	**Value**	**OR**	**SE**	***t* value**	***P***
Gender	0.318	1.374	0.216	1.469	0.142
Age	−0.015	0.985	0.023	−0.671	0.502
Surgery history	−0.333	0.716	0.479	−0.697	0.486
Contact lenses >2 h per day	−0.293	0.746	0.304	−0.963	0.336
**Total sleep duration**	−0.253	0.776	0.122	−2.069	**0.039**
**Total VDT using duration**	0.187	1.205	0.044	4.242	**<0.001**
Duration of each VDT use	0.046	1.047	0.078	0.594	0.553
Mild|Moderate	−0.366	0.693	1.262	−0.290	0.772
Moderate|Severe	2.406	11.085	1.273	1.890	0.059

*The bold values indicate statistically significant values of p < 0.05*.

## Discussion

### Prevalence of VDTs

Through this survey, it was found that the prevalence of VDTs was high in the students of these two medical colleges in high-altitude regions, Kunming, reaching 73.2%. In 2016, Ling and others of Central South University found the prevalence of VDTs to be 57.3% in a questionnaire study of 1,350 general enrolled college students (14). Compared with this survey, the prevalence of VDTs in medical students in these two colleges in Kunming was significantly increased compared with the above studies. There are two reasons for consideration. Firstly, the respondents of this paper are medical students. As a reserve force of a special profession, medical students have been well-known for their high academic pressure and heavy tasks, but there is still a lack of attention to their health. In the process of learning in today's society, the use of VDT in various aspects such as learning and communication has also increased significantly. With the advent of the post-epidemic era, the learning mode of the Internet has been widely used and popularized, and in the era of information explosion, the ensuing series of adverse effects is increasingly being taken into account. Secondly, the subjects of this paper were all from college students who had been studying in Kunming, a high-altitude area, for more than 1 year, while Zhongnan University is located in Hunan, China, a non-high-altitude area, which may lead to a higher prevalence of VDTs in the subjects of this paper due to the possible aggravation of dry eye symptoms at high altitude. The symptoms of VDTs, such as blurred vision and headaches, have significantly affected the quality of life and work of modern people, especially young people, and this has led to a significant increase in the number of patients seen for VDTs, making us sensitive to the fact that VDTs are a series of syndromes that cannot be ignored in our lives, that significantly affect the quality of life, and that may cause irreversible damage. Patients with these syndromes should be given more attention in our medical activities. In our medical activities, more attention should be paid to patients with such syndromes.

### Influencing Factors of VDTs

The results of this questionnaire showed that total daily sleep duration as well as total daily VDT use were the influencing factors of VDTs. The severity of eye discomfort symptom was significantly negatively related to the increased total sleep duration and it was significantly positively related to the increased VDT use duration. This is consistent with the findings by Fjaervoll et al. (15) and others. They similarly concluded that prolonged VDT use and shortened sleep duration are closely related to the emergence of VDTs (16–18). Prolonged use of VDT is the cause of eye discomfort, which may include inappropriate operation methods, incorrect eye habits and so on. Most people may place the monitor high for comfort when using electronic products, which results in gazing at the screen with the line of sight facing upward, resulting in large exposure of the cornea to air and accelerated water evaporation, which to some extent affects the quality of the tear film, thus leading to a shorter tear film rupture time and causing ocular dryness and discomfort (3, 19). At the same time, due to long-term staring at the electronic screen, the significant reduction of blink rate and blink amplitude can likewise affect the tear film break-up time and the distribution of tear film, leading to the development of eye discomfort (20). This was investigated in a study by Cardona et al. (21) with a sample of 25 healthy young VDT users and found that a highly demanding visual task had an effect on flicker rate, flicker amplitude, and tear film integrity. The reason for the significant decrease in blink rate, on the other hand, may be due to the high level of concentration when operating electronic products, which produces excessive gaze, resulting in reduced lacrimal gland secretory capacity and uneven tear distribution, which can only be compensated by increasing blink frequency (22, 23).

### Relationship Between VDTs and Duration of VDT Use, Eye Surgery, Wearing Contact Lenses, and Gender

The results of multivariate regression analysis in this paper showed that the duration of each VDT use, whether contact lenses were worn for more than 2 h, and gender were not sufficient to affect the occurrence of eye discomfort symptoms. This may be related to the possibility that long-term use of contact lenses may cause damage to the corneal nerve tissue, manifested in part as reduced sensitivity to discomfort symptoms. It has also been clearly reported in a study of Malcolm that the use of contact lenses decreases corneal sensitivity (24). In addition, the gender indicator may be affected by the uneven proportion of male and female students enrolled in medical schools. At the same time, the sex ratio of the observational study participants included in this paper is also unbalanced, so the sex in this paper is not included in the risk factor analysis of whether it is the risk factors for the disease of video terminal syndrome of medical students in high altitude areas.

As VDT gradually becomes an increasingly inseparable part of people's daily life, especially in the context of VDT users gradually tending to be younger, it is important to study the characteristics of the disease in the population used and the health hazards that may be complicated during use to ensure the healthy development of each of us. This investigation and analysis of VDT use and ocular symptoms of high-altitude regions in medical students has certain representativeness, which is helpful to understand the VDT exposure of contemporary college students and young people, so as to formulate reasonable prevention and control measures for this current situation and reduce or avoid property and health losses caused by incorrect use of VDT.

## Data Availability Statement

The original contributions presented in the study are included in the article/supplementary material. Further inquiries can be directed to the corresponding author.

## Ethics Statement

The studies involving human participants were reviewed and approved by the Ethics Committee of Human Research in The First People's Hospital Yunnan Province. Participants provided their verbal informed consent.

## Author Contributions

BL and ZC planned and designed the study. SJ, YW, ZL, and DZ contributed to data collection, data analysis, and data interpretation. BL played a leading role in writing the manuscript. ZC revised the manuscript. All authors read and approved the final manuscript.

## Funding

This study was financially supported by Medical Reserve Talents Training Program of Yunnan Provincial Health Commission (H-2018024), Inoue Expert Workstation in Yunnan Province (202005AF150030).

## Conflict of Interest

The authors declare that the research was conducted in the absence of any commercial or financial relationships that could be construed as a potential conflict of interest.

## Publisher's Note

All claims expressed in this article are solely those of the authors and do not necessarily represent those of their affiliated organizations, or those of the publisher, the editors and the reviewers. Any product that may be evaluated in this article, or claim that may be made by its manufacturer, is not guaranteed or endorsed by the publisher.
